# CTLA4-Ig + Anti-IL-6/IL-6R Treatment Results in Long-term Allograft Survival and Function in Highly HLA-sensitized Patients

**DOI:** 10.1097/TXD.0000000000001952

**Published:** 2026-06-02

**Authors:** Stanley C. Jordan, Sindhu Chandran, Noriko Ammerman, Edmund Huang, Jun Shoji, Xiaohai Zhang, Alice Peng, Bongha Shin, David Thomas, Reiad Najjar, Sanjeev Kumar, Peter Heeger, Ashley Vo

**Affiliations:** 1 Comprehensive Transplant Center, Cedars-Sinai Medical Center, West Hollywood, CA.

## Abstract

**Background.:**

Highly HLA-sensitized (HS) patients are at increased risk for cell-mediated rejection (CMR)/antibody-mediated rejection (AMR) posttransplant and require intense immunosuppression to prevent these events. HS patients who experience calcineurin inhibitor (CNI) neuro/nephrotoxicity have few options as conversion to CNI-free regimens with cytotoxic T-lymphocyte-associated antigen (CTLA4)-Ig may risk donor specific antibody (DSA) rebound, CMR, and AMR. This remains an unmet medical need. Anti-interleukin (IL)-6/IL-6R treatment is useful in treating AMR and CMR and preventing rejection in HS patients after transplantation (tx). Here, we examined the utility of combining CTLA4-Ig with anti-IL-6/IL-6R treatment in 4 HS patients with CNI toxicity.

**Methods.:**

Four HS patients (3 with calculated panel reactive antibodies >97% at tx and 1 who developed chronic-active AMR (c-aAMR) 14 y post-tx were evaluated. Two patients, 100% calculated panel reactive antibody and +flow crossmatch at transplantation, were desensitized with plasma exchange + clazakizumab (anti-IL-6) and maintained on clazakizumab post-tx and transitioned from CNI to CTLA4-Ig. Two patients treated with anti-IL-6R (tocilizumab) were transitioned to CTLA4-Ig at 2 and 11 y post-tx. Patients were monitored for renal function (RF), DSA, de novo DSA (dn-DSA), immune cell subsets, and patient and graft survival.

**Results.:**

Clazakizumab and tocilizumab treated patients showed improvements in RF, with elimination of existing DSAs, except for 1 patient and no dn-DSAs (>6 y). We also saw reductions in CD8^+^ T_EFF/MEM_, T_FH_, B_MEM_, and NK cells post-CTLA4-Ig + Anti-IL-6/IL-6R treatment. T_REG_ and B_REG_ cell populations also increased, suggesting, but not confirming, possible immune modulation.

**Conclusions.:**

Combining CTLA4-Ig + anti-IL-6/IL-6R appears to offer benefits in sustaining RF, DSA reduction, possibly immune cell modulation, and prevention of dn-DSA. Importantly, CNI toxicity abated in all patients.

## INTRODUCTION

Belatacept is a fusion protein of the Fc fragment of human IgG1 with the extracellular domain of cytotoxic T-lymphocyte-associated antigen (CTLA4). The fusion protein, (CTLA4-Ig), limits T-cell costimulation by competitive antagonism of the CD28^-^CD80/CD86 signaling pathway.^1^ Despite early enthusiasm for a calcineurin inhibitor (CNI)-free immunosuppression regimen, that also reduced de novo donor specific antibody (dn-DSA) production,^2,3^ real-world experience revealed high rates of rejection, likely due to costimulatory resistant (CSR) CD8^+^T-effector/memory cells (CD8^+^T_EFF_/_MEM_) and a negative impact on T_REG_ induction,^4,5^ which limited therapeutic implementation. Subsequent studies utilizing CTLA4-Ig with T-cell depletion using thymoglobulin and alemtuzumab failed to prevent early rejection as well.^6^ Clearly, these concerns are amplified in HLA-sensitized (HS) kidney transplant recipients because only low immunologic risk patients were included in trials utilizing belatacept for de novo immunosuppression. However, CNI toxicity is often seen in HS patients due to increased exposure aimed at limiting dn-DSA development. Here, CTLA4-Ig showed important benefits compared with cyclosporine A in limiting dn-DSA development.^3^

We previously reported on CTLA-Ig use for CNI toxicity in HS patients.^7^ In our study, among HS and non-HS patients, there was no difference in rejection-free, patient, or graft survival after conversion to belatacept over a 5-y observation period. However, conversion during the first-year posttransplant resulted in lower rejection-free survival for HS recipients. Importantly, studies of CTLA4-Ig treatment in mouse heart transplant models have shown that CTLA4-Ig treatment alone is not sufficient to prevent early rejections, likely due to injury induced by CD8^+^T_EFF_/_MEM_ cells which are CSR because of lack of CD28 expression.^8,9^ Investigators have also shown that CTLA4-Ig treatment alone inhibits T_REG_ expansion, further promoting risk for rejection. Understanding CSR CD8^+^T_EFF_/_MEM_ cell and other effectors responsible for mediation of cell-mediated (CMR) and antibody-mediated rejection (AMR) could help address this major deficiency of CTLA4-Ig therapy.

Interleukin-6 (IL-6) is a multifunctional cytokine critical for T-follicular/helper cells (T_FH_) development, B-cell maturation, IgG class switch, and adaptive plasma cell immune responses.^10,11^ A review of data investigating IL-6/IL-6R signaling pathway inhibition in animal models and human studies suggests it likely offers benefits in modulation of T_FH_, B-cell and plasma cell responses, lowering pathogenic IgG antibodies in addition to inhibition of CD8^+^T_EFF_/_MEM_ cells while enhancing T_REG_ populations.

Here, we explored combining CTLA4-Ig with anti-IL-6/IL-6R treatment in HS patients at risk for AMR who also developed CNI toxicity.

## MATERIALS AND METHODS

### Patients

We describe in this report the background, clinical course and immunologic findings in four HS patients (3 with calculated panel reactive antibody (cPRAs) >97% at transplant and one who developed c-aAMR 14 y posttransplantation) who were maintained on a novel CNI-free regimen combining CTLA4-Ig with anti-IL-6/IL-6R treatment.

Patients were monitored for renal function (RF), DSA, dn-DSA, immune cell subsets (3 of 4 patients), and patient and graft survival. The study was conducted in accordance with the Declaration of Helsinki with the ethics guidelines based on federal regulations and the Common Rule. It was approved by the institutional review board at Cedars-Sinai Medical Center (CSMC) in Los Angeles, California. The 4 patients evaluated are from the CSMC Kidney Transplant Program, and all study activities occurred at CSMC after enrolled patients provided written informed consent.

### Donor-specific Anti-HLA Antibody Testing

HLA antibodies were detected by the single antigen bead-based assay (One lambda, Los Angeles, CA). Donor HLA-specific antibodies were assessed throughout treatment as previously described.^12^ Detection of DSAs were only reported with mean fluorescence intensity (MFI) >2500. All samples collected were treated with EDTA to improve accuracy of DSA detection and eliminate nonspecific binding to assay beads. All samples were analyzed at the CSMC HLA and Immunogenetics Laboratory (American Society of Histocompatibility & Immunogenetics-certified) using Luminex testing platforms.

### Immune Cell Phenotyping Studies

Immune cell phenotyping studies were performed on 3 of 4 patients after >4 y on CTLA4-Ig + anti-IL-6/IL-6R treatment and compared with values established in normal individuals. This including assessment of CD8^+^T_EFF_/_MEM_ (CD45RA^−^, CD45RO+, CD8^+^), T_fh_ (CD4^+^, CXCR-5^+^/ICOS^+^, PD1^+^) T_REG_ (CD4^+^, CD25^+^, CD127^LOW^, FoxP-3+), B_MEM_ (CD19^+^, CD27^+^, CD38^LOW^), B_reg_ (CD19^+^, CD24^Hi^, CD38^Hi^, CD138^−^), plasmablast (CD19^+^, CD27^+^, CD38^+^, CD138^−^), and NK cell (CD56^+^) populations using flow cytometry analysis that was performed at CSMC Transplantation and Immunology Laboratory (Clinical Lab Improvement Act- and College of American Pathology-certified).^12^ Briefly, sodium heparinized peripheral blood cells were first stained with antibodies to T-cell, B-cell, and NK-cell surface and intracellular antigens, including those described earlier. After lysing red blood cells followed by permeabilization, the cells were stained with antibodies to intracellular targets. A complete compendium of immune cell phenotyping targets and normal values is provided in **Figure S1 (SDC**, https://links.lww.com/TXD/A865).

## RESULTS

### Case Reports

#### Case 1: CNI Toxicity and Chronic AMR With De Novo HLA DSA

**FIGURE 1.**
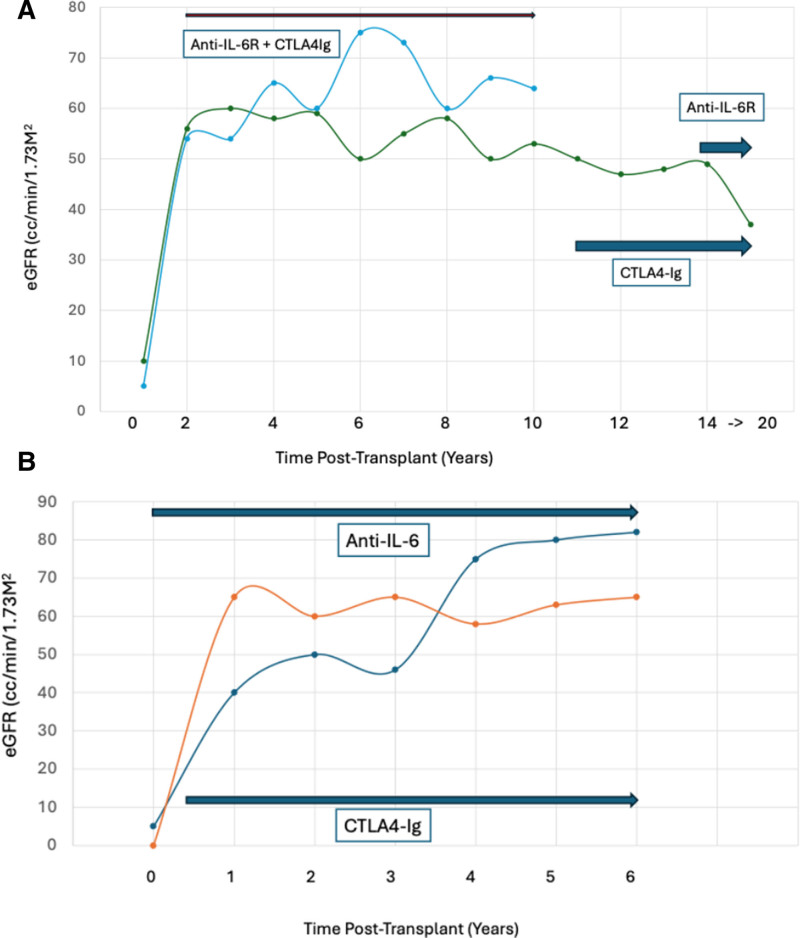
A, eGFR for 2 patients treated with CTLA4-Ig and anti-IL-6R. B, eGFR for 2 patients treated with anti-IL-6 and CTLA4-Ig. eGFR was calculated using CKD-EPI 2021. CKD-EPI, chronic kidney disease epidemiology collaboration equation for eGFR measurment, CTLA4, cytotoxic T-lymphocyte-associated antigen; IL, interleukin.

A 53-y-old man, nonsensitized with end stage renal disease (ESRD) secondary to reflux nephropathy and chronic pyelonephritis underwent a living unrelated donor renal transplant. He received basiliximab induction and was maintained on cyclosporine, mycophenolate mofetil (MMF) and prednisone. A kidney transplant biopsy 7 y posttransplant for elevated serum creatinine and proteinuria showed borderline AMR and TCMR, and evolving chronic rejection. He was treated with steroids, rituximab, and IVIg. DSA was detected against HLA DQA1*05 (MFI > 10 000) that has remained persistently elevated. Repeat biopsy 4 y later showed active AMR and findings suggesting chronic CNI nephrotoxicity. Cyclosporine was discontinued, and belatacept was initiated. A biopsy 3 y later showed predominantly chronic transplant glomerulopathy. At this point tocilizumab, a humanized IgG1 monoclonal antibody to the IL-6 receptor was added at a dose of 8 mg/kg IV monthly. Since then, the patient has been maintained on belatacept, tocilizumab, 360 mg daily MMF, and prednisone 5 mg daily. DSA remained stable on this regimen. He has now been on a CNI-free regimen for >9 y with stable graft function (estimated glomerular filtration rate [eGFR] ~37 mL/min/1.73 m^2^, urine protein/creatinine ratio [UPC] 0.3 g/g) now >21 y posttransplant. No infectious complications occurred over the period of follow-up (Figure 1A). Of note, recent declines in eGFR from 48 mL/min/1.73 m^2^ are likely because of glucagon like peptide-1 agonist therapy with dehydration and weight loss of 81 lbs.

#### Case 2: CNI Toxicity and Chronic AMR With no HLA DSA

A 43-y-old woman with ESRD secondary to pre-eclampsia and failed kidney transplant had cPRA 99.97%. She underwent HLA desensitization with plasmapheresis, IVIg, and rituximab. She received a flow cytometric crossmatch positive (FCXM+) deceased donor kidney transplant with pretransplant DSA to HLA A32 (MFI 7500). She received alemtuzumab induction and was maintained on tacrolimus, MMF, and prednisone. DSA became undetectable at 2 mo posttransplant. A kidney transplant biopsy 2 y posttransplant for proteinuria (urine protein: creatinine 2.2 g/g) showed chronic AMR and chronic CNI nephrotoxicity. HLA DSA remained undetectable. Tocilizumab (8 mg/kg IV monthly) was started. Belatacept was added 3 mo later, and tacrolimus was tapered off >2 mo. Since then, her regimen has consisted of belatacept, tocilizumab, 180 mg BID MMF, and prednisone 5 mg PO daily. She has been on this CNI-free regimen for 6 y 6 mo with stable graft function (eGFR 64 mL/min/1.73 m^2^, UPC 0.4 g/g). The patient experienced 4 infection-related hospitalizations, for pneumonia (COVID-19) and graft pyelonephritis, over the follow-up period. She is currently doing well (Figure 1A).

#### Case 3: CNI Toxicity Without Rejection

A 59-y-old woman with ESRD secondary to reflux nephropathy and failed kidney transplant had cPRA 100%. She enrolled in a clinical trial (NCT03380962) testing the safety and tolerability of clazakizumab, a humanized IgG1 monoclonal antibody to IL-6 for HLA desensitization,^12^ and underwent treatment with plasmapheresis, IVIg, and clazakizumab (6 doses of 25 mg SC monthly) as part of the study protocol. She received a FCXM^+^ deceased donor kidney transplant shortly thereafter with pretransplant DSA to HLA DR52 (MFI 7500) and HLA DR17 (MFI 2500). She received alemtuzumab induction and was maintained on tacrolimus, MMF, and prednisone. Clazakizumab continued posttransplant as per the study protocol. DSA became undetectable at 1-mo posttransplant. A surveillance kidney transplant biopsy at 6 mo showed CNI nephrotoxicity and no acute rejection. No HLA DSA was detected. Belatacept was added and tacrolimus was tapered off >4 mo. Since then, her regimen has consisted of belatacept, clazakizumab, azathioprine 25 mg every other day (because of intolerance to MMF) and prednisone 5 mg daily. She has been on a CNI-free regimen for 6 y and 7 mo with stable graft function (eGFR 68 mL/min/1.73 m^2^, UPC 0.14 g/g). Over the follow-up period, there were 2 infection-related hospitalizations, for graft pyelonephritis and groin abscess, respectively (Figure 1B).

#### Case 4: CNI Toxicity Without Rejection

A 48-y-old man with ESRD secondary to polycystic kidney disease and two prior failed kidney transplants had cPRA 100%. He was enrolled in NCT03380962 and underwent HLA desensitization with plasmapheresis, IVIg, and clazakizumab (6 doses of 25 mg SC monthly). Seven months later, he received an FCXM^+^ deceased donor kidney transplant with a pretransplant DSA to DP17 (MFI 17500). He received alemtuzumab induction and was maintained on tacrolimus, MMF, and prednisone. Clazakizumab was resumed following a transplant as per study protocol. Posttransplant, DSA against HLA DP17 persisted. A kidney transplant biopsy at 3 mo posttransplant for elevated serum creatinine showed acute CNI nephrotoxicity and no features of rejection. Belatacept was added and tacrolimus was tapered off >5 mo. Since then, he has been maintained on belatacept, clazakizumab, MMF 180 mg BID, and prednisone 5mg daily. DSA abated and became undetectable at 2 y posttransplant. He has been on a CNI-free regimen for 6 y and 2 mo with stable graft function (eGFR 85 mL/min/1.73 m^2^, no proteinuria). No infectious complications occurred during follow-up (Figure 1B).

### Immune Cell Phenotyping

**FIGURE 2.**
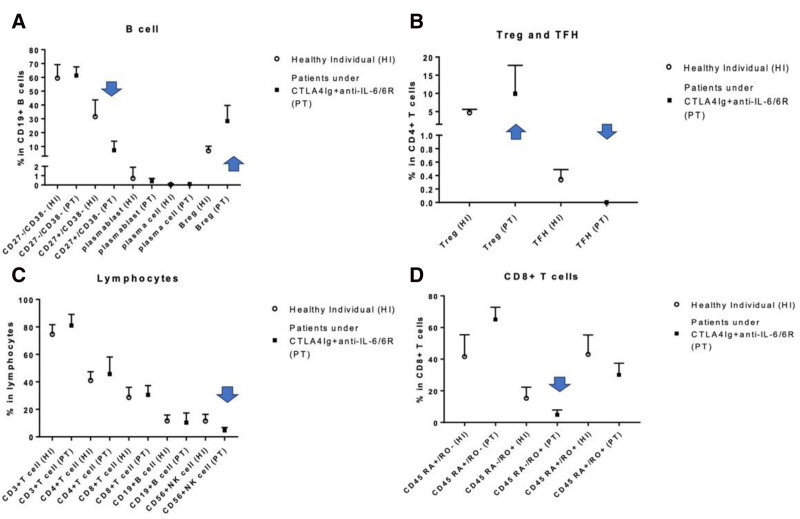
These figures show the impact of CTLA4-Ig + anti-IL-6/IL-6R treatment on circulating immune cell subsets compared with normals. A, The impact of CTLA4-Ig +anti-IL-6/IL-6R treatment on circulating B-cell populations. Here, reductions in circulating B_MEM_ (CD19^+^, CD27^+^, CD38^LOW^) cells and increased B_REG_ (CD19^+^, CD24^Hi^, CD38^Hi^, CD138^−^) cells were seen. Circulating plasmablast (CD19^+^, CD27^+^, CD38^+^, CD138^−^) also trended lower than what was seen in normals. B, The impact of CTLA4-Ig + anti-IL-6/IL-6R cell on T_REG_ (CD4^+^, CD25^+^, CD127^LOW^, FoxP-3+) and T_FH_ (CD4^+^, CXCR-5^+^/ICOS^+^, PD1^+^) populations. Here, treatment resulted in increased T_REG_ populations and inability to detect circulating T_FH_ cells compared with normals. C, Little impact on lymphocyte subsets except for reductions in circulating NK cells. D, The impact of therapy on circulating CD8^+^ T-cell subsets. Importantly, CTLA4-Ig + anti-IL-6/IL-6R treatment was associated with increased CD45RA^+^, CD45RO-CD8^+^ (naive CD8^+^ T cells) and reduced CD45RA^−^, CD45RO^+^, CD8^+^ (CD8^+^T_EFF_/_MEM_) cell populations. Data were obtained from 12 to 16 normal individuals for controls and 3 of 4 patients. CTLA4, cytotoxic T-lymphocyte-associated antigen; IL, interleukin.

As part of this evaluation, we examined immune cell phenotypes in CTLA4-Ig + anti-IL-6/IL-6R treated patients and compared them with normal individuals. Here, data presented represent a single analysis at >4 y after initiation of therapy in 3 of 4 patients. One patient was not analyzed because of lack of proximity to the medical center. Overall, we noted little impact of CTLA4-Ig + anti-IL-6/IL-6R on lymphocyte subsets except for reductions in circulating NK cells. However, analysis of circulating CD8^+^ T-cell subsets showed that CTLA4-Ig + anti-IL-6/IL-6R treatment was associated with increased CD45RA^+^, CD45RO-CD8^+^ (naive CD8^+^ T cells) and reduced CD45RA^−^, CD45RO^+^, CD8^+^ (CD8^+^T_EFF_/_MEM_) cell populations. The impact of CTLA4-Ig + anti-IL-6/IL-6R on T_REG_ (CD4^+^, CD25^+^, CD127^LOW^, FoxP-3^+^) and T_FH_ (CD4^+^, CXCR-5^+^/ICOS^+^, PD1^+^) cell populations showed an increased T_REG_ population and inability to detect circulating T_FH_ cells compared with normals. CTLA4-Ig + anti-IL-6/IL-6R treatment resulted in reductions in circulating B_MEM_ (CD19^+^, CD27^+^, CD38^LOW^) cells and increased B_REG_ (CD19^+^, CD24^Hi^, CD38^Hi^, CD138^−^) cells. We also saw that circulating plasmablast (CD19^+^, CD27^+^+, CD38^+^+, CD138^−^) populations trended lower than normal. Data are shown in Figures 2A–D.

## DISCUSSION

To the best of our knowledge, this is the first report of sensitized kidney transplant recipients being successfully maintained on a long-term CNI-free immunosuppressive regimen containing belatacept and either clazakizumab or tocilizumab. The tolerability and durability of this approach is supported by the stability of RF and lack of rejection for >6 y in all patients. In addition, these patients demonstrated reduction in posttransplant DSA (2 patients). The therapy was overall well tolerated. However, 1 patient treated with tocilizumab did develop severe COVID pneumonia from which she has recovered. Importantly, signs and symptoms of CNI toxicity are abated in all patients. One patient, now 21 y posttransplant treated late with CTLA4-Ig and tocilizumab, showed recent declines in eGFR 48–37 mL/min/1.73 m^2^. This, we think, is because of excessive weight loss and dehydration because of glucagon like peptide-1 agonist therapy. We have advised him to stop this medication.

Although preliminary and with limited sample size and frequency that prevent a more robust analysis, immune cell phenotyping indicated that there may be important modifications in circulating immune cells that aid in establishing immune modulation rather than immune suppression. Critical to this assessment are 2 reports. First, Chandran et al^13^ reported on the use of tocilizumab to treat patients with renal allograft inflammation. In a controlled trial, the investigators observed that tocilizumab treatment was associated with reduced graft inflammation and increases in T_REG_ cells with reductions in γ-interferon and IL-17 compared with controls. Vo et al^12^ presented data on clazakizumab treatment for desensitization. In an open-label study of 20 HS patients, clazakizumab resulted in reductions of pretransplant HLA antibodies that allowed all 20 patients to receive HLA-incompatible transplants. Importantly, clazakizumab dosing was maintained posttransplant and resulted in no dn-DSAs and reductions in preexisting DSAs at 1-y posttransplant. In addition, patients were found to have increased T_REG_ and B_REG_ cell populations at 6-12M posttransplant. In the patients maintained on clazakizumab and tocilizumab after CTLA4-Ig treatment and elimination of CNIs, we found that reductions in CD8^+^ T_EFF/MEM_, T_FH_, B_MEM_, and NK cells post-CTLA4-Ig + anti-IL-6/IL-6R treatment. T_REG_ and B_REG_ cell populations also increased posttreatment.

Our findings are consistent with data from preclinical animal models. In a mouse model of incompatible heart transplantation, Zhao et al^8^ examined the role of IL-6 in mediation of rejection and tolerance. Transplants in IL-6-deficient mice did not prolong survival beyond nontreated controls; however, was associated with increased T_REG_ cells in the allografts. This is compared with ~34-d survival with CTLA4-Ig alone. Importantly when CTLA4-Ig treatment was given to the IL-6 (−/−) mice transplants, >100-d survival was seen and associated with reductions in differentiation of effector cells and increased intragraft T_REG_. These investigators also showed that anti-IL-6 + CTLA4-Ig gave >100 d graft survival as well. They concluded that inhibition of IL-6, when combined with strategies that inhibit Th1 responses (ie, CTLA4-Ig), has a synergistic effect on the promotion of allograft acceptance. Thus, targeting the effects of IL-6 production could represent a pathway to improve costimulation blockade-based strategies by compensating for CSR to promote allograft acceptance and tolerance. This is the first article to recognize the importance of anti-IL-6 as a means for compensating for CSR T-cell mediation of rejection with CTLA4-Ig alone. More recently Muckenhuber et al^9^ reported on the limitations of CTLA4-Ig (belatacept) in transplantation which is associated with an increased risk of T cell–mediated rejection, despite induction with antithymocyte globulin (ATG). Here, the investigators showed why ATG fails to prevent costimulation blockade-resistant rejection and how this can be overcome. ATG did not prevent graft rejection in a murine heart transplant model using CTLA4-Ig and induced a proinflammatory cytokine environment. Although ATG improved the balance between regulatory T_REG_ and effector T cells in the spleen, the cardiac allografs continued to show inflammation with high IL-6 levels. Importantly, anti-IL-6 treatment alleviated graft inflammation, increased intragraft T_REG_, and increased intragraft IL-10. The investigators conclude that IL-6 inhibition together with ATG allowed CTLA4-Ig therapy to achieve long-term, rejection-free heart allograft survival. However, this beneficial effect was terminated when T_REG_ cells were depleted. These authors also concluded that combining ATG with IL-6 blockade prevents costimulation blockade-resistant rejection, which could eliminate the barriers to early use of CTLA4-Ig based therapies posttransplantation. Finally, Yabou et al^14^ reported that addition of peritransplant tocilizumab to CTLA4-Ig significantly prolonged graft survival and reduced γ-interferon and tumor necrosis factor-α producing CD4 and CD8 effector T cells cells in an MHC-mismatched rhesus monkey kidney transplant model. Overall, these data suggest that the combination of costimulatory and IL-6 blockade is synergistic at promoting tolerance.

Recent data from our group and others have shown an important therapeutic benefit of tocilizumab in treatment of c-aAMR. Nobel et al^15^ retrospectively studied kidney transplant patients with c-aAMR or microvascular inflammation (MVI) without (DSA) and C4d deposition (MVI^+^ DSA^−^ C4d^−^). The study was performed in 2 European centers and included 64 patients who received tocilizumab as first-line therapy. eGFR and DSA were assessed 1 y before and after tocilizumab initiation. The eGFR trajectory significantly decreased after tocilizumab treatment (−1.2 ± 0.2 versus 0.03 ± 0.2 mL/min/1.73 m^2^/mo pre- versus post-tocilizumab, respectively; *P* = 0.001). The percentage of patients with DSA decreased from 63.9% to 38.9% (*P* < 0.001), Patient survival was 98.4%, and graft survival was 93.7% at 1 y. The authors concluded that first-line tocilizumab therapy for c-aAMR or MVI^+^ DSA^−^C4d^−^ is associated with an improvement of eGFR trajectories, reduced DSA numbers, and MFI and histological inflammation in glomeruli. These data suggest a potential benefit of tocilizumab for treatment of c-aAMR and MVI^+^ DSA^−^ C4d^−^.

Our group also recently reported on the treatment of c-aAMR and MVI^+^ DSA^−^ C4d^−^ renal transplant patients treated with tocilizumab.^16^ In a retrospective observational study, we investigated the association of monthly tocilizumab infusions with the trajectory of (eGFR) among kidney transplant patients with histologic features of c-aAMR. 85 adult kidney transplant patients with histologic features of c-aAMR treated with monthly tocilizumab 8 mg/kg intravenous infusions were identified. We compared eGFR trajectories 12M before and 12M after tocilizumab initiation. The eGFR declined at a rate of −0.70 mL/min/1.73 m^2^/mo (95% CI, −1.03 to −0.36) for 1-y preceding tocilizumab initiation and stabilized after 1 y treatment to a slope of −0.07 (−0.35 to 0.21) mL/min/1.73 m^2^/mo (*P* = 0.002). Examination of DSA^+^ status showed 14 of 65 DSA^+^ patients (22%) no longer had DSA at 1 y. We observed one graft loss and 2 deaths, both COVID-19-related at 12 mo after treatment onset. This study suggests that monthly treatment with tocilizumab, may stabilize allograft function among kidney transplant patients with c-aAMR and MVI^+^ DSA^−^ C4d^−^ patients.

Both studies are important since both show a stabilizing effect of tocilizumab on patients with c-aAMR and MVI^+^ DSA^−^ C4d^−^.The mechanism(s) of this benefit may be related to reduction of inflammatory cell activation and infiltration and DSA reduction, but may relate to modulation of injury pathways induced by DSAs in endothelial cells that initiate IL-6 production eventually translating to increased fibrosis which can be modulated by anti-IL-6/IL-6R.^17,18^

The recognition over the last 2 decades that that chronic AMR plays a dominant role in late renal allograft loss has led to increased efforts to augment maintenance immunosuppression, especially in those who are highly sensitized to HLA and/or have posttransplant DSA. However, these patients already have a high cumulative burden of immunosuppression due to previous failed transplants. Indeed, in some cases, intolerance to CNI may have contributed to previous allograft losses. Such patients present a therapeutic conundrum and an unmet need and could benefit from novel CNI-free regimens. Although preliminary, and limited in scope, we feel our data are encouraging, especially due to the durability of this therapeutic intervention. However, larger, prospective clinical trials of this regimen with systematic analysis of RF, biopsy tissue, and immune cell subsets are needed to fully understand the safety, efficacy, and mechanistic impacts of this approach.

## Supplementary Material


