# Supplementary figures and images for: CTLA4-Ig + Anti-IL-6/IL-6R Treatment Results in Long-term Allograft Survival and Function in Highly HLA-sensitized Patients
Source: Transplant Direct. 2026 Jun 2;12(7):e1952. doi: 10.1097/TXD.0000000000001952 (PMC13232908; doi:10.1097/TXD.0000000000001952)

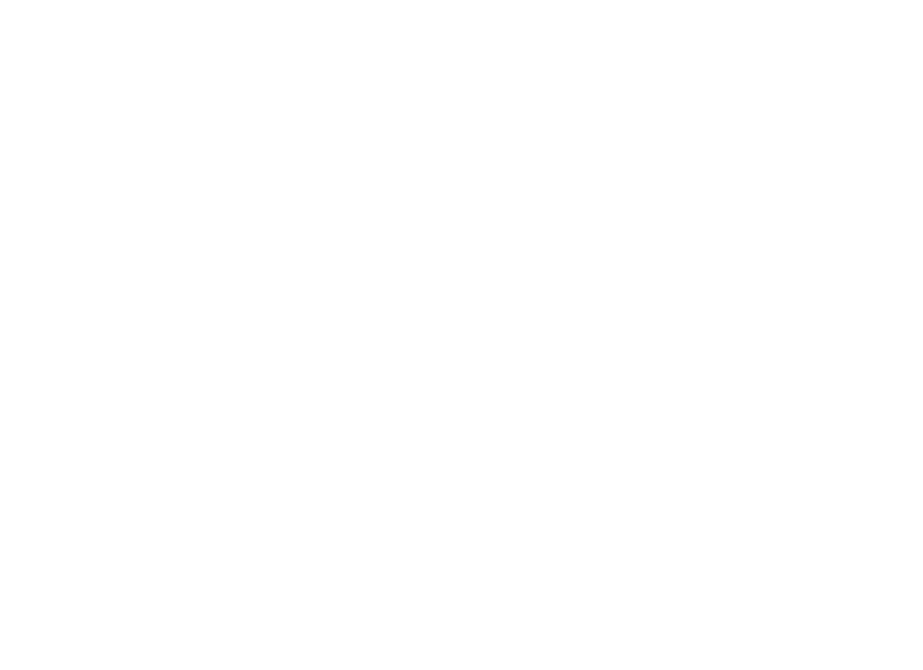

Supplement: Supplementary file 1 [file txd-12-e1952-s001.tif]
